# Osteosarcoma follow-up: chest X-ray or computed tomography?

**DOI:** 10.1186/s13569-017-0067-5

**Published:** 2017-02-14

**Authors:** Anna Paioli, Michele Rocca, Luca Cevolani, Eugenio Rimondi, Daniel Vanel, Emanuela Palmerini, Marilena Cesari, Alessandra Longhi, Abate Massimo Eraldo, Emanuela Marchesi, Piero Picci, Stefano Ferrari

**Affiliations:** 1 Chemotherapy Unit, Istituto Ortopedico Rizzoli, via Pupilli, 1, 40136 Bologna, Italy; 2 General Surgery Unit, Istituto Ortopedico Rizzoli, via Pupilli, 1, 40136 Bologna, Italy; 3 Department of Orthopaedic Oncology, Istituto Ortopedico Rizzoli, via Pupilli, 1, 40136 Bologna, Italy; 4 Diagnostic and Interventional Radiology, Istituto Ortopedico Rizzoli, via Pupilli, 1, 40136 Bologna, Italy; 5 Department of Pathology, Istituto Ortopedico Rizzoli, via di Barbiano, 1/10, 40136 Bologna, Italy

**Keywords:** Osteosarcoma, Follow-up, Chest X-ray, Chest computed tomography

## Abstract

**Background:**

In patients with relapsed osteosarcoma, the surgical excision of all metastases, defined as second complete remission (CR-2), is the factor that mainly influences post-relapse survival (PRS). Currently a validated follow-up policy for osteosarcoma is not available, both chest X-ray and computed tomography (CT) are suggested for lung surveillance. The purpose of this study is to evaluate whether the type of imaging technique used for chest surveillance, chest X-ray or CT, influenced the rate of CR-2 and prognosis in patients with recurrent osteosarcoma.

**Methods:**

Patients up to 40 years with extremity osteosarcoma enrolled in consecutive clinical trials and treated at the Rizzoli Institute from 1986 to 2009 were identified. Only patients who had lung metastases alone as first pattern of recurrence were considered for the analysis. The rate of CR-2, overall survival (OS) and PRS were the end-points of the study.

**Results:**

The median follow-up was 47 months (1–300), 215 patients were eligible. Lung metastases were detected by chest X-ray in 100 (47%) patients, by CT in 112 (52%) and by symptoms in 3 (1%). CR-2 rate was 60% for patients followed by X-rays and 88% for those followed by CT (p < .0001). 5-year PRS was 30% (95% CI 21–39) in the X-ray group and 49% (95% CI 39–59) in the CT group (p = .0004). 5-year OS was 35% (95% CI 26–44) in the X-ray group and 60% (95% CI 51–70) in the CT group (p = .004).

**Conclusions:**

A follow-up strategy with chest CT leads to a higher rate of CR-2 and significantly improves PRS and OS in osteosarcoma, compared to chest X-ray.

## Background

High-grade osteosarcoma is the most frequent primary bone tumor, with a peak of incidence in the second decade of life [1, 2]. The addition of multi-agent chemotherapy to surgery alone significantly improved survival rate, however, almost 40% of patients with localized disease relapse [3]. Recurrences usually occur within 3 years after the end of treatment, but late relapses, even after more than 10 years, are reported [4]. The most frequent site of metastasis is the lung (more than 80% of cases), local recurrences occur in less than 10% of cases [5–7].

Post-relapse survival (PRS) after distant recurrence is poor [5–7]. The complete removal of all metastases, defined as a second complete surgical remission (CR-2), is the main prognostic factor for PRS and relate to long survival [8–16].

Other prognostic factors related to a better PRS are the site of relapse (lung vs others), the number of lung nodules (less than two) and a relapse free interval (RFI) of more than 2 years [5–7].

The role of chemotherapy in recurrent osteosarcoma is not yet well defined, but it has recently been emphasized in patients with a short relapse-free interval or in those who cannot achieve a complete surgical remission [3, 5, 6].

Osteosarcoma surveillance programs should be able to detect recurrence when complete removal of all known tumor sites is still feasible. Currently, an evidence-based follow-up policy is not available. International guidelines stress the importance of an intensive follow-up program focusing on the chest and on the primary tumor site, particularly for the first 4–5 years [3, 17]. A radiological follow-up is recommended for the chest, with X-ray or CT scan. Chest CT scan is more sensitive and sensible than chest X-ray in detecting lung metastases, but is burdened by higher radiation exposure and costs [18–20]. In clinical practice, follow-up programs vary in the different centers, both in terms of schedule and in terms of imaging techniques in use [21]. Whether the type of follow-up influences prognosis in patients with osteosarcoma is still debated.

In order to evaluate if the technique of chest surveillance (X-ray or CT) influence CR-2 rate and survival, we performed a retrospective cohort analysis of patients treated in a single Institution.

## Patients and methods

Patients enrolled in clinical trials performed at the Rizzoli Institute between 1986 and 2006 were included in the analysis. Details of these studies have been published. IOR/OS-2 enrolled patients from 1986 to 1989 [22], IOR/OS-3 from 1990 to 1993 [23], IOR/OS-4 from 1993 to 1995 [24], Pilot ISG/OS from 1996 to 1997 [25], ISG/SSG-1 from 1997 to 2000 [26] and ISG/OS-1 from 2001 to 2006 [27].

Briefly, protocols included patients aged up to 40 years with localized high-grade osteosarcoma of the extremity. The strategy of treatment was based on delayed surgery after primary chemotherapy. The neo-adjuvant schemes of treatment were mainly based on methotrexate, doxorubicin, cisplatin, ±ifosfamide. From our database we selected for the analysis the patients who relapsed and between these, those who had lung metastases alone at time of the first recurrence. Clinical charts were reviewed. Data collection was in accordance with the local ethical committee standards. A statement on consent to use the data for scientific purposes and publication was obtained from all patients. The imaging techniques used for chest surveillance changed over the study period. From 1986 to 1995 [22–24] follow-up was performed with chest X-ray, from 1996 to 2000 [25, 26] both chest X-ray and CT scan were used. Since 2001 patients were followed up only by CT [27]. When chest X-rays was used, follow-up schedule was every 2 months for the first 2 years, every 3 months the 3rd year, and then every 6 months. A confirmatory CT scan of the chest was performed in case of suspected nodules. Starting from 1996 all patients who completed chemotherapy underwent CT of the chest, whereas those who were already followed up by X-rays continued with the same technique. When chest CT was used, follow-up schedule was every 3 months for the first 2 years, every 4 months in the 3rd and 4th year, and then every 6 months. All the patients were evaluated in a multidisciplinary team at the time of recurrence of the disease. The surgical approach foresaw lateral thoracotomy and manual palpation of the whole lung. In case of bilateral lesions, a contemporary surgery or two-staged surgery was performed. A wedge resection was performed when possible. Surgical staplers were used only in selected cases.

The rate of patients who achieved CR-2, overall survival (OS), and post relapse survival (PRS) were the end-points of the study. For each patient, date of the first surgical remission (CR-1), date of the first recurrence, pattern of recurrence, the imaging technique used for chest follow-up (X-ray or CT), relapse-free interval (RFI), number and size of lung nodules, laterality, treatment at recurrence were collected. Relapse free interval (RFI) was calculated from the date of CR-1 to first relapse. Post relapse survival (PRS) was calculated from the date of first relapse to date of death or last follow-up. Overall survival (OS) was calculated from the date of CR-1 to date of death or last follow-up. Survival curves were compared by Kaplan and Meier. Chi square and *t* test were used for comparison between groups when appropriate.

## Results

**Fig. 1:**
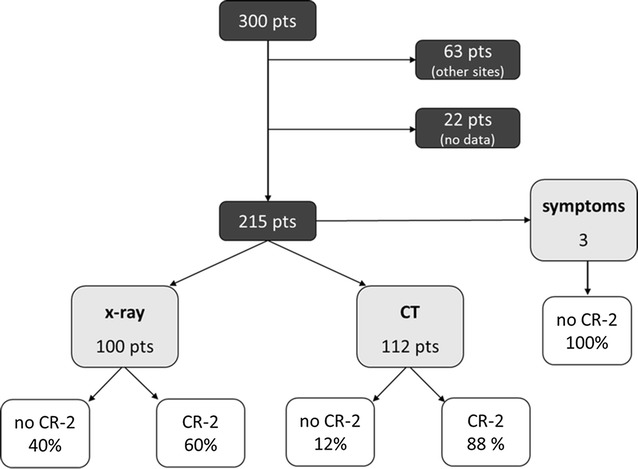
Patients eligible for the analysis, method of lung metastases detection and rates of CR-2. *pts* patients, *X*-*ray* chest X-ray, *CT* chest computed tomography, *CR*-*2* second complete surgical remission

Over the study period, 300 patients who recurred with lung metastases were identified. Lung metastases were associated with other recurrences in 63 patients, incomplete data were found in 22 patients. The remaining 215 patients were eligible for the analysis (Fig. 1). Median age at time of the first relapse was 15 years (range 6–43 years), 130 (60%) patients were male and 85 (40%) female.

Median RFI was 25 months (3–136 mos). Overall, 178 (83%) patients underwent surgery and 159 (74%) achieved a CR-2 status. All patients who did not reach CR-2 died. The 3- and 5-year PRS was 45% (95% CI 38–52) and 39% (95% CI 32–46) respectively, and the median PRS was 22 months (range 2–280 mos). With a median follow-up of 47 months (1–300 mos), OS was 64% (95% CI 58–71) at 3 years and 47% (95% CI 40–54) at 5 years.

Lung metastases were detected by chest X-ray in 100 (47%) patients, by CT scan in 112 (52%) and by symptoms in 3 (1%) patients. These last 3 patients, all followed-up by chest X-ray, were excluded from the analysis, none achieved a CR-2 and they all died of the disease.

Comparing the two groups of patients we observed no difference in terms of age, sex and site of the primary disease, while the rate of good responder patients was higher in the X-ray group compared to the CT group (Table 1). At first recurrence, the number of lung nodules did not differs among the two groups, while the incidence of bilateral lung metastases was higher and nodules were larger when lung relapses were detected by chest X-ray (Table 2). We observed a shorter median RFI and a higher rate of patients with a RFI of *<*2 years in the CT group.

**Table 1:** Patient and disease features according to imaging technique used for follow-up

	X-ray 100 pts	CT 112 pts	p
Age (years)			
Range	6–43	6–42	.7
Median	15	16	
Sex			
F	42 (42%)	42 (38%)	.5
M	58 (58%)	70 (62%)	
Site			
Femur	49 (49%)	56 (50%)	.9
Tibia	30 (30%)	32 (29%)	
Humerus	16 (16%)	19 (17%)	
Other	5 (5%)	5 (4%)	
Histological response	(90 pts)	(94 pts)	.02
Good	62 (69%)	49 (52%)	
Poor	28 (31%)	45 (48%)	

*pts* patients, *X*-*ray* chest X-ray, *CT* computed tomography

**Table 2:** Pattern of lung relapse according to imaging technique used for follow-up

	X-ray 100 pts	CT 112 pts	p
RFI			
Median (months)	28.4	22.3	.01
<2 years (pts)	49 (49%)	72 (64%)	.02
Laterality			
Monolateral	63 (63%)	88 (79%)	.01
Bilateral	37 (37%)	24 (21%)	
n. nodules (162 pts)	84 pts	78 pts	.4
1	52 (62%)	47 (60%)	
2–5	23 (27%)	21 (27%)	
>5	9 (11%)	10 (13%)	
Size (112 pts) (cm)	54 pts	58 pts	.03
<2	20 (37%)	37 (64%)	
2–5	26 (48%)	19 (33%)	
≥5	8 (15%)	2 (3%)	

*pts* patients, *X*-*ray* chest X-ray, *CT* computed tomography, *RFI* relapse free interval

At the time of recurrence, the use of chemotherapy was mainly based on high-dose ifosfamide. As reported in Table 3 the percentage of patients who received chemotherapy at recurrence did not differ in the chest-X-ray group compared with the CT group (p = .5). Most patients treated with chemotherapy underwent surgery, 24 in the X-ray group and 34 in the CT group. In both groups only 7 patients were treated preoperatively.

**Table 3:** Treatment and incidence of second complete surgical remission (CR-2) according to imaging technique used for follow-up

	X-ray 100 pts	CT 112 pts	p
Surgery			
Yes	73 (73%)	105 (94%)	<.0001
No	27 (27%)	7 (6%)	
1°line Chemo	(89 pts)	(104 pts)	.5
Yes	31 (35%)	41 (39%)	
No	58 (65%)	63 (61%)	
CR-2	60 (60%)	99 (88%)	<.0001

*pts* patients, *X*-*ray* chest X-ray, *CT* computed tomography, *Chemo* chemotherapy, *CR*-*2* second complete surgical remission

Patients were surgically treated in two Centers (Unit of General Surgery of the University of Modena and Unit of General Surgery of the Rizzoli Institute) by three surgeons B.A., M.R., C.S. All patients underwent lateral thoracotomy and manual palpation of the lung. In order to evaluate the whole lung, no patient was treated with video-assisted thoracoscopic resection. Up to 2000, bilateral wedge resection was performed in 22% of the patients, monolateral wedge resection in 57% of the patients, lobectomy with or without wedge resection in 21%. In the most recent years the percentage of monolateral wedge resection was 70%, bilateral wedge resection was 15%, lobectomy with or without wedge resection 15%.

Patients underwent surgery and achieved a CR-2 more frequently in the CT group as compared to the X-ray group (Table 3). CR-2 rate was 60% in patients followed up by X-ray, whereas it was 88% in those followed up by CT scan (p < .0001). We observed that the rate of patients who underwent surgery without achieving a CR-2 was higher in the chest X-ray group compared with CT group, 18 and 6% respectively (p = .01).

From 1996 to 2000 both X-ray and CT were used for chest surveillance. Seventy-five patients were included in this period, 19 were followed up by CT and 56 by X-ray. A CR-2 was obtained in all the patients followed with CT and in the 57% of patients followed by X-ray (p = .001).

**Fig. 2:**
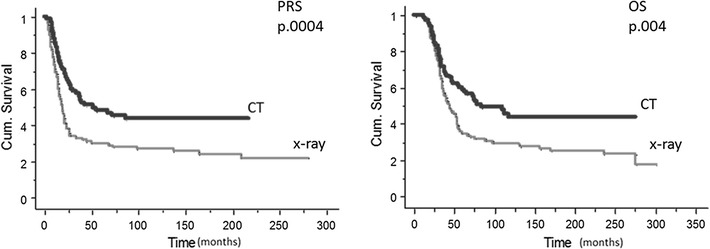
Post relapse survival (PRS) and Overall survival (OS) at Kaplan and Meier analysis according to imaging technique used for follow-up. *PRS* post relapse survival, *OS* overall survival, *X*-*ray* chest X-ray, *CT* chest computed tomography

The difference in terms of CR-2 translated in a significant difference in terms of PRS and OS between the two groups (Fig. 2). The 3- and 5- year PRS was 33% (95% CI 33–42) and 30% (95% CI 21–39) in the chest X-ray group and 58% (95% CI 49–68) and 49% (95% CI 39–59) in the CT scan group (p = .0004) (Table 3). Overall survival at 3 years was 58% (95% CI 48–68) in the X-ray group and 72% (95% CI 63–80) in the CT scan group, at 5-years it was 35% (95% CI 26–44) and 60% (95% CI 51–70) in the two groups, with a difference that was statistically significant. (p = .004) (Table 4).

**Table 4:** Post relapse survival and overall survival at 3 and 5 years according to imaging technique used for follow-up

	X-ray 100 pts	CT 112 pts
PRS		
3-year PRS	33% (95% CI 33–42)	58% (95% CI 49–68)
5-year PRS	30% (95% CI 21–39)	49% (95% CI 39–59)
OS		
3-year OS	58% (95% CI 48–68)	72% (95% CI 63–80)
5-year OS	35% (95% CI 26–44)	60% (95% CI 51–70)

*pts* patients, *PRS* post relapse survival, *OS* overall survival, *CT* computed tomography, *95% CI* 95% confidence interval

## Discussion

Results from our analysis show that the routinely use of chest CT scan, compared with X-ray, in the follow-up of osteosarcoma patients leads to a higher rate of second complete surgical remission (CR-2) and, consequently, to a significant benefit both in terms of PRS, and in terms of OS.

One of the main aim of oncology follow-up is early detection of recurrence, especially when effective strategy of treatment can be offered. We restricted the study population on patients who had metastases confined to the lung to emphasize better the potential benefit related to early diagnosis with regard to the follow-up programs. It is well known that this group of patients has a better probability of survival compared to that reported for those with multiple metastatic sites.

Relapse free interval of less than 2 years usually relate with a worse prognosis [5–7]. In our series we observed a higher rate of patients with RFI <2 years but a significantly better PRS and OS in the CT group compared with the X-ray group. The median RFI was shorter when patients were followed-up by CT, suggesting that the better prognosis observed in this group was probably due to the early diagnosis of recurrence, which led patients to be treated with surgery with radical intent more frequently, with a higher rate of CR-2.

The higher rates of necrosis after neo-adjuvant chemotherapy reported in the X-ray group compared with the CT group were probably related with the use of intraarterial infusion of cisplatin in most patients up to 1995 [22–24].

It is widely accepted that CT scan is superior to X-ray for the detection of lung nodules, but is under discussion whether the routine use of CT for chest surveillance can influence prognosis in patients with osteosarcoma. Data available in literature mainly comes from retrospective studies and only one prospective randomized study, which included both soft tissue and bone sarcomas.

It is interesting to note that in one study on soft tissue sarcomas where chest X-ray was used for follow-up, in 21 (37%) of 57 patients, lung metastases were detected when the patients had symptoms of lung involvement [28].

Similar results were reported in a more recent paper [29] where patients with high-grade primary bone or soft tissue sarcoma of the extremities followed-up by chest X-ray were included. Thirty-seven percent of the 90 patients with bone sarcomas who developed pulmonary metastases were detected outside the follow-up program, 13 were symptomatic. Overall, only nine (10%) patients survived after recurrence.

A retrospective analysis on a heterogeneous group of 174 patients with low and high grade soft tissue sarcomas has been reported [30]. Most were followed-up by chest X-ray. Only in 9 of 22 patients who developed isolated and asymptomatic lung metastases surgical resection was feasible whereas for the remaining patients the extent of the pulmonary disease did not allow metastasectomy.

A study that compared CT and X-ray of the chest in soft tissue sarcomass did not find any difference of survival related to the modalty of follow-up for the whole study population [31]. Nevertheless, focusing on the 54 patients who recurred with lung metastases, the 4-year survival rate after detection of pulmonary metastasis was 0% in the chest X-ray cohort whereas it was 31.6% in the chest CT cohort (p < .05). Interestingly, as shown in our analysis, also in this study, unilateral lesions of smaller size were more frequently detected in CT group, leading to a higher rate of surgical complete remission.

The same group recently published results from a small retrospective study on high grade extremity osteosarcomas, both localized and metastatic [32]. The imagining technique recommended for chest surveillance was X-ray, CT scan was planned at the end of the treatment, in case of suspicious finding or in case of lung metastasis at diagnosis [32]. Twenty-two patients relapsed with lung metastases alone, 3 were detected by symptoms, 10 by X-ray and 9 by CT. The rate of patients surgically treated with curative intent was higher in the X-ray group (9/10) compared to CT group (6/9), as was number of patients remain relapse free, 5 in the X-ray group and 2 in the CT group. According to the authors, this difference is probably related to the higher baseline risk of relapse in patients followed with CT.

Only one randomized prospective trial on follow-up programs for patients with sarcoma of the extremities has been published [33]. Follow-up schedule (every 3 and 6 months) and chest imaging (chest X-ray and CT scan) were compared in a two-by-two factorial design. Authors reported that 3-year overall survival was 67% in the CT scan group and 66% in the X-ray group and concluded that the study demonstrated the non-inferiority of chest X-ray as compared to CT scan. Many criticisms can be addressed to this study. Firstly the heterogeneity of the sample, that included in the same analysis patients with soft tissue sarcomas and bone sarcomas, high and low grade and primary and recurrent sarcomas. Furthermore no data regarding histology of the bone sarcomas were given. It is well known that all these different entities have a different clinical behavior and a different response to treatments. For these reasons, the results obtained cannot be applicable to a single histotype and should not be considered conclusive. In our opinion, it is important to stress that the end point of the Indian study was the overall survival at 3 years. A rather short follow-up period considering that the available lines of chemotherapy treatment and the aggressive surgical approach can prolong post relapse survival in bone and soft tissue sarcomas [3, 4, 34, 35]. Also in our study if we considered overall survival, the difference was less apparent at 3 years (58 vs 72%), but at 5 years it reached a statistically significant (35 vs 60%).

We are aware of the radiation risks related to the routine use of CT for chest surveillance, particularly in children and young adults [36–39]. On the other hand, a 25% gain in terms of OS at 5 years as reported in our analysis indicated that the benefit related to the use of CT exceeded the risks of second malignancies associated [40].

A limit of our study is its retrospective design, and the long period of observation. A change in surgical techniques over the years could be a potential bias influencing results. However surgical procedures that allow the removal of metastases including a small amount of normal tissue around, like wedge resections, were available since early 1980s. The vast majority of the patients in our study, including patients who relapsed in the 1980s, were treated with wedge resections, monolateral or bilateral. It is important that the surgeon could evaluate the whole lung, in order to detect unrecognized nodules, for this reason no patient was treated with thoracoscopic resection [41]. Moreover, in the group of patients included when both X-ray and CT were used for chest surveillance, the higher rate of CR-2 related with the use of CT compared with X-ray was confirmed. The higher rate of patients who underwent surgery without achieving CR-2 reported in the X-ray group did not seem due to the availability of different surgical techniques, but rather to the delay of the diagnosis of recurrence and the higher incidence of large and bilateral lung metastases observed in this group.

Surgery was the treatment of choice at the time of first recurrence, however some patients in our series were treated with chemotherapy. The rate of patient treated with first line chemotherapy did not differ among the two group of patients. On the other hands, it is questionable whether chemotherapy at the time of recurrence has an impact on post relapse survival. In a previous paper including patients evaluated in the present analysis, we could not observe any benefit from chemotherapy in patients surgically free of disease [6]. A strength of our study is that we included a large and homogenous series of patients, treated and followed-up in a single Institution. All patients had high grade localized osteosarcoma at diagnosis and lung metastases alone at first recurrence. The surgical directions did not changed over the study period. At the time of recurrence all the patients were evaluated in a multidisciplinary way together with the surgeon and, if indicated, they were treated by the same surgical team. In the group of patients followed-up with X-ray, in case of pathological findings, a confirmatory CT was performed. At the same way, in case of uncertain diagnosis when very small size nodules were detected in the CT group of patients, a new confirmatory CT was performed after 1 or 2 months, in order to evaluate the nodule growth. In the management of small lung nodules, when usually the resectability is not a problem, it is important to assess their behavior, in order to avoid overtreatment.

## Conclusions

In conclusion, in patients with osteosarcoma of the extremity a follow-up strategy based on chest CT allows a higher rate of second complete remission and significantly improves prognosis with a higher probability of post relapse and overall survival when compared to surveillance based on chest X-ray.

## Abbreviations

CR-2: second complete surgical remission
PRS: post relapse survival
OS: overall survival
CT: chest computed tomography
Chemo: chemotherapy
X-ray: chest X-ray
pts: patients
CR-1: first complete surgical remission
RFI: relapse free interval
95% CI: 95% confidence interval
Mos: months
CR-1: first complete surgical remission
