# Erratum to: Osteosarcoma follow-up: chest X-ray or computed tomography?

**DOI:** 10.1186/s13569-017-0073-7

**Published:** 2017-05-03

**Authors:** Anna Paioli, Michele Rocca, Luca Cevolani, Eugenio Rimondi, Daniel Vanel, Emanuela Palmerini, Marilena Cesari, Alessandra Longhi, Massimo Eraldo Abate, Emanuela Marchesi, Piero Picci, Stefano Ferrari

**Affiliations:** 1 Chemotherapy Unit, Istituto Ortopedico Rizzoli, via Pupilli, 1, 40136 Bologna, Italy; 2 General Surgery Unit, Istituto Ortopedico Rizzoli, via Pupilli, 1, 40136 Bologna, Italy; 3 Department of Orthopaedic Oncology, Istituto Ortopedico Rizzoli, via Pupilli, 1, 40136 Bologna, Italy; 4 Diagnostic and Interventional Radiology, Istituto Ortopedico Rizzoli, via Pupilli, 1, 40136 Bologna, Italy; 5 Department of Pathology, Istituto Ortopedico Rizzoli, via di Barbiano, 1/10, 40136 Bologna, Italy

## Erratum to: Clin Sarcoma Res (2017) 7:3 DOI 10.1186/s13569-017-0067-5

It was noticed that there was an error in the author list, upon publication of the original article [1]. The ninth author should be listed as Massimo Eraldo Abate.
